# An Intelligent Monitoring Network for Detection of Cracks in Anvils of High-Press Apparatus

**DOI:** 10.3390/s18041142

**Published:** 2018-04-09

**Authors:** Hao Tian, Zhaoli Yan, Jun Yang

**Affiliations:** 1 Institute of Acoustics, Chinese Academy of Sciences, Beijing 100190, China; tianhao13@mails.ucas.ac.cn (H.T.); jyang@mail.ioa.ac.cn (J.Y.); 2 Key Laboratory of Noise and Vibration Research, Institute of Acoustics, Chinese Academy of Sciences, Beijing 100190, China; 3 University of Chinese Academy of Sciences, Beijing 100049, China

**Keywords:** intelligent monitoring network, crack detection, convolutional neural network, spatial pyramid pooling layer (SPP-Layer), class imbalance problem, adaptive weighted algorithm for loss function

## Abstract

Due to the endurance of alternating high pressure and temperature, the carbide anvils of the high-press apparatus, which are widely used in the synthetic diamond industry, are prone to crack. In this paper, an acoustic method is used to monitor the crack events, and the intelligent monitoring network is proposed to classify the sound samples. The pulse sound signals produced by such cracking are first extracted based on a short-time energy threshold. Then, the signals are processed with the proposed intelligent monitoring network to identify the operation condition of the anvil of the high-pressure apparatus. The monitoring network is an improved convolutional neural network that solves the problems that may occur in practice. The length of pulse sound excited by the crack growth is variable, so a spatial pyramid pooling layer is adopted to solve the variable-length input problem. An adaptive weighted algorithm for loss function is proposed in this method to handle the class imbalance problem. The good performance regarding the accuracy and balance of the proposed intelligent monitoring network is validated through the experiments finally.

## 1. Introduction

At present, synthetic diamonds are produced in a high-temperature and high-pressure environment formed by electric heating and the hydraulic press in the high-press apparatus. In this process, the anvil cracks may be caused by material fatigue as a result of the alternating high temperature and pressure. A cracked anvil will eventually fracture as the cracks grow, and the concomitant sudden loss of pressure may damage other anvils, causing safety accidents. Thus, the crack monitoring of anvils is necessary to ensure the high-press apparatus works reliably and efficiently.

The study of fatigue issues began in the middle of the nineteenth Century. Through a lot of experimental explorations, a wealth of knowledge has been accumulated. The S-N curve, which indicates the fatigue performance and fatigue limit, were first proposed by Wholer in the 1860s. Bauschinger put forward the concept of stress-strain hysteresis loop in 1884, and in 1945, Miner presented the Palmgrem theory of linear cumulative damage and the Miner-Palmgren theory. Until now, these theories were widely used [1]. The development of fracture mechanics in the 1950s further promoted the study of the fatigue crack propagation law and failure control. In order to simulate the process of crack growth, various fatigue crack propagation rate models have been established, such as the Paris model [2], the Walker model [3], and the Forman model [4], which have been successfully applied in engineering. However, the fatigue failure involves the multiple actions of the load, the formation and expansion of material defects, the influence of the working environment, etc. The complexity of the problem is obvious. Therefore, many problems of fatigue failure have not been fully understood and fundamentally resolved. On the other hand, except for the fatigue life prediction, the real-time monitoring of fatigue cracks is also important in practice.

The acoustic emission (AE) is the most commonly used method in fatigue crack monitoring. It is generated when the material cracks. As the AE signal contains the information of damage degree of the material [5,6], the characteristics of the AE signal are often used to determine the working state of the mechanical equipments. Yan proposes the nested power spectrum centroid and modified power spectrum variance as features of the pulse sound signal to detect the cracks in the anvil of large-volume cubic high-pressure apparatus [7]. In Zhou’s work, for monitoring the railway vehicle axle fatigue crack, an Elman neural network is adopted to classify the AE signals using the feature vector consisting of energy entropy, the energy distribution ratio, and interval average energy [8]. The time-frequency manifold features of the AE signals are extracted to interpret different bearing conditions and are used for bearing fault diagnosis by He [9]. Wen uses the non-stationary features in the time and frequency domain of the AE signal, which is processed by wavelet packet decomposition and the empirical mode decomposition for tool condition classification with the ReliefF method [10]. Al-Ghamd identifies the presence and size of a defect on a radially loaded bearing with AE technique and proves that AE offers earlier fault detection and provides an indication of the defect size [11]. Shahidan uses the AE wave descriptors, including AE amplitude, rise time, and average frequency, to identify the crack patterns in concrete beams [12]. Although this method has achieved some results, there are still lots of defects.

**Figure 1:**
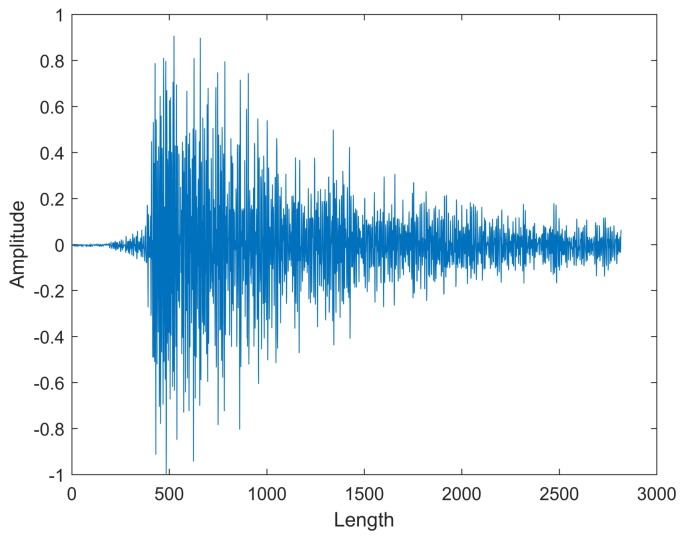
The pulse sound signal.

The AE wave is a solid-elastic wave that can only be detected by touch sensors. Since the anvils are working in a high temperature environment, the AE sensors attached to the anvils are liable to fail as the couplant dries rapidly. In addition, as many as six sensors are needed for each high-pressure apparatus. Thus, the pulse sound wave, which is excited by the AE wave, is detected using one microphone (a kind of non-contact sensor) in this paper. A typical pulse sound signal is shown in Figure 1. Generally, the process of extracting feature parameters and identifying faults mainly relies on manual work, which is time-consuming and laborious. Additionally, previous studies have shown that the algorithm developed for one type of high-press apparatus is ineffective for another type of high-press apparatus, which greatly limits its applications. In order to realize data-driven automatic feature extraction and signal identification, an intelligent monitoring network is established. It can not only apply to the monitoring high-press apparatus, but also to other mechanical equipment.

**Figure 2:**
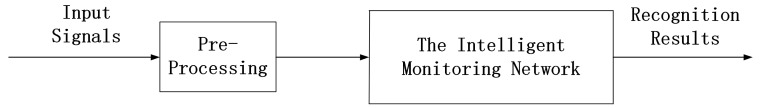
The flow chart of the algorithm.

A complete set of algorithms for monitoring the crack of anvils of the high-press apparatus is presented in this paper. The sound pulse signals are first extracted from original signals, which contain information related to the working status of high-press apparatus. Then, the operation condition of the high-press apparatus is identified by processing the pulse sound signals through the monitoring network (the flow chart of the algorithm is shown in Figure 2). The monitoring network is based on the convolutional neural network, which is widely applied in image recognition and is modified to meet the specific requirements of high-press apparatus monitoring. Finally, the experiment results are given and discussed. The method proposed in this paper realizes real-time monitoring of the carbide anvils of the high-press apparatus.

## 2. Pre-Processing

**Figure 3:**
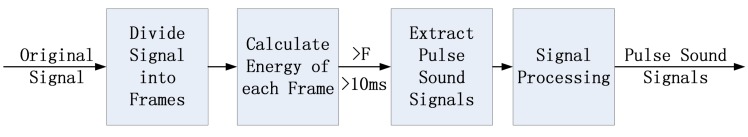
The Pre-Processing of Original Signal.

The Pre-Processing works on the basis that the pulse sound signal has higher energy than the background noise. The original data is divided into non-overlapping frames at first step, and then the energy of each frame is calculated. The pulse sound signal is assumed to start once the frame energy exceeds a threshold F and to continue until the energy drops below this threshold. The pulse sound signals less than 10 ms are ignored to avoid possible electromagnetic interference. The steps of Pre-Processing are shown in Figure 3.

The threshold value (F) is obtained by the following method: A certain number of frames of background noise signal are selected, and the average frame energy of these signals is gained, which is denoted by $F_{noise}$. Then, F is calculated by Equation (1).

(1) $$F=\beta \cdot F_{noise}$$

in which β is a coefficient greater than 1 and set to 1.4 as an empirical value in this article.

To deal with the possible continuous double sound pulse, the variation of frame energy is utilized. Considering that the later pulse sound signal is usually superposed over the attenuation stage of previous one, there is a valley of frame energy ($Min_{Mid}$) between the two actual pulse sound signals with energy peaks of $Max_{For}$ and $Max_{Lat}$. If $Min_{Mid}$ is less than α(α<1) times the minimum of two peaks ($Min_{Mid}<\alpha \cdot min\left\{Max_{For},Max_{Lat}\right\}$), it is separated into two pulse sound signals; otherwise, it is regarded as a single pulse sound signal. α is called the double pulse extraction parameter.

## 3. The Intelligent Monitoring Network

**Figure 4:**
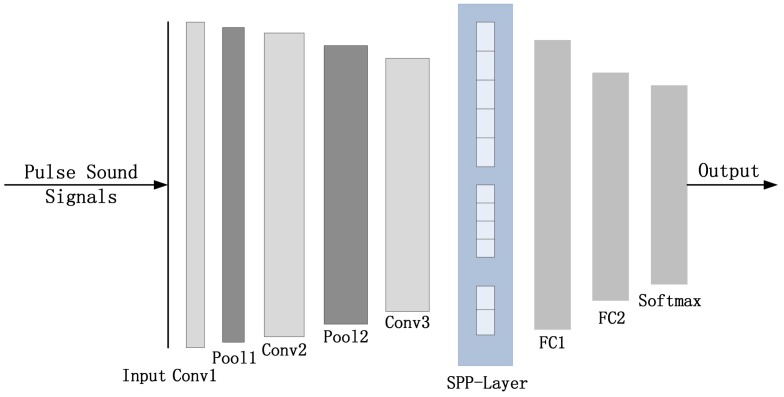
The Structure of the Monitoring Network.

The pulse sound signals are analyzed and recognized by the proposed intelligent monitoring network. It is an improved convolutional neural network and consists of three convolutional layers, two max-pooling layers, two fully connected layers, a spatial pyramid pooling layer (SPP-Layer), and a Softmax classifier. The specific structure is illustrated in Figure 4.

### 3.1. Convolutional Network

A convolutional network, which is composed of three convolutional layers and two pooling layers, is adopted to learn the representations of the pulse sound signal. A series of one-dimensional convolutions followed by ReLU activation function is used to process the pulse sound signals. The convolutional network is well-suited for the signals in this paper for several reasons. Firstly, the convolutional network has a powerful capability of feature extraction proven by its applications in signal processing. Secondly, the convolutional network makes it practical to stack layers, which enables the convolutional network to detect higher-level features. What is more, the recognition of pulse sound signals of different complexity under various working conditions can be realized by adding or reducing the convolutional layers without changing the structure of other parts [13,14,15,16].

The structure parameters of convolutional network are displayed in Table 1, which include the lengths of inputs, the number of filters, the size of filters, and the stride of filters in each layer.

### 3.2. Variable Length Input/Output

**Figure 5:**
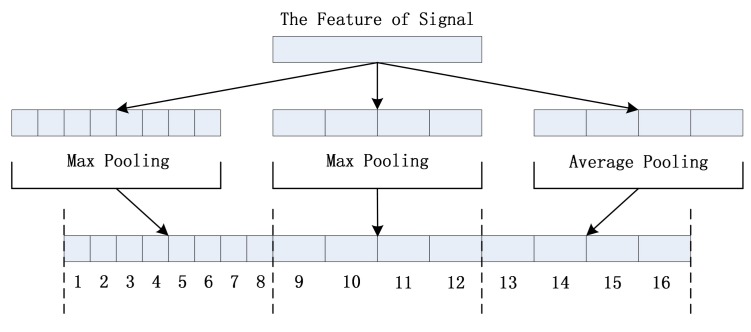
The Workflow of SPP-Layer.

Since the time lengths of pulse sound signals excited by the crack growth are variable, the monitoring network must handle the variable-length inputs. As the convolutional network includes only convolutional layers and pooling layers, it can work with input of different lengths. To make the output layer work with variable length input as well, a SPP-Layer is added following the convolutional network [17]. The variable length input is transformed into the fixed length output via SPP-Layer. The workflow of the SPP-Layer is shown in Figure 5. The input of length N is divided into floor(N/8) and floor(N/4) frames. Then, the Max pooling operation is done on floor(N/8) frames and floor (N/4) frames, and the average pooling is done on floor(N/4) frames, respectively. The results of pooling operations are combined at last to form the fixed length output.

The output is processed through two fully connected layers, the dimension of which is reduced. At the end, a Softmax classifier is used to serve as the final output layer for providing the identification of each pulse sound signal. The change in the length of the input is illustrated in Table 2.

### 3.3. The Adaptive Weighted Algorithm for Loss Function

The ratio of normal signal samples to fault signal samples has a significant impact on the classification performance during the training of the intelligent monitoring network. In fact, the classification with minor proportion of training samples gives a poor recognition rate. While, in practice, the anvils of high-press apparatus are less prone to failure, the fault signal samples are in the minority within the training dataset. Given this condition, an adaptive weighted algorithm for loss function is proposed in this paper to ensure the balance of the monitoring network. The algorithm is defined as blow:

1. The training sample expansion of fault signals is obtained firstly using the methods of amplitude reversal, time reversal, white noise adding, and random copping [18,19,20].
2. The power spectrum centroid and the 1st nested power spectrum centroid of all the fault signal samples in the training dataset are calculated using the method proposed in Yan’s work [1], denoted by $F_{0}$, $F_{1}^{0}$, $F_{1}^{1}$.
3. In the parameter space with the dimensions of $F_{0}$, $F_{1}^{0}$, $F_{1}^{1}$, the sum of the Euclidean distances from the fault signal sample to its k nearest fault signal neighbors (k=5 in this article) is calculated, which is denoted by $R_{sum}$. Then, the loss function of fault signal samples is given a corresponding weight to increase the penalty for misclassifying. The ratio of the normal signal samples to the fault signal samples in the training dataset is defined as ρ, then the weight value can be calculated by Equation (2).

(2) $$\rho_{i}\;=\;\frac{max\left\{R_{sum}\right\}-R_{sum}\left(i\right)}{max\left\{R_{sum}\right\}-minth\left\{R_{sum}\right\}}\cdot \rho +0.5\cdot \rho ,$$

in which ρ is supposed to be larger than two in general, $R_{sum}\left(i\right)$ is the sum of the distances from the i-th fault signal sample to its k nearest fault signal neighbors, and $\rho_{i}$ is the weight of the loss function of the i-th fault signal sample.

This algorithm mainly reflects the distribution of the fault signal samples by Euclidean distance. The small value of $R_{sum}$ indicates that the fault signal sample locates close to the cluster center, which should play a major role in the network training and be assigned a large weight value, or else a small weight should be given. On the other hand, in order to avoid the network imbalance caused by oversize $\rho_{i}$, four common algorithms are used to realize the training sample expansion of fault signals in step 1.

## 4. Experiments and Analysis

### 4.1. Original Data

The original data used in this paper is collected continuously for about 13 h from high-press apparatuses in factory, of which the sampling rate is 131,072 Hz and is recorded in mat format.

### 4.2. Data Processing

In the experiments, the frame length and the double pulse extraction parameter α in Pre-Processing are set to 256 and 0.4, respectively. A total of 4392 pulse sound signals are extracted, among which 124 signals are fault signals and 4268 signals are normal signals. Each pulse sound signal is labeled by hand. Initially, 30 fault signal samples and 1000 normal signal samples are selected randomly as the testing dataset. Then, the rest of the fault signal samples are processed by the time reversal and amplitude reversal, white noise adding (SNR 20), and random copping respectively. Finally, a total of 470 fault signal samples and 3268 normal signal samples are gained as the training dataset.

The training of the monitoring network is implemented on Tensorflow. Through the tests and analyses, the Adam optimizer and weighted cross entropy loss function are adopted, and batch size of 100 produces good results. The network initializes all the weights to zero-mean Gaussian noise with a standard deviation of 0.02, and all the bias are set to 0.1. The training ended after 500 iterations.

The operation mentioned above is repeated 10 times independently. The results consist of recognition accuracy on the whole samples (Acc), the normal signal samples (Acc-N), and the fault signal samples (Acc-F), which are as shown in Table 3. Compared with the results of Yan’s algorithm [7], the recognition accuracy on fault signals increases from 81% to 91% on the basis of maintaining the recognition accuracy on normal signals. The monitoring network shows good performance in terms of accuracy and balance.

### 4.3. Experiment Analysis

**Figure 6:**
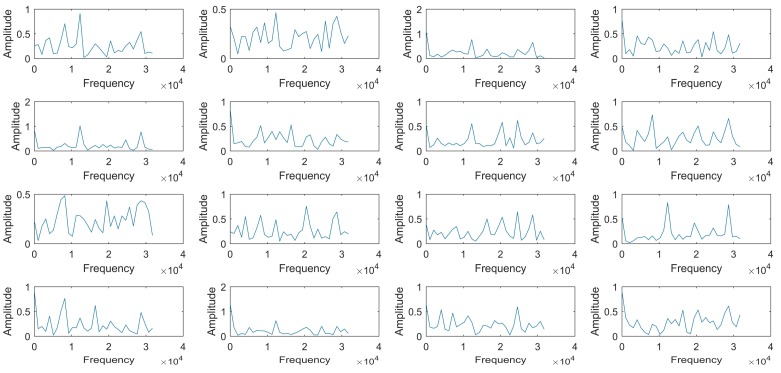
The amplitude spectrum of learned filters in Conv1.

**Figure 7:**
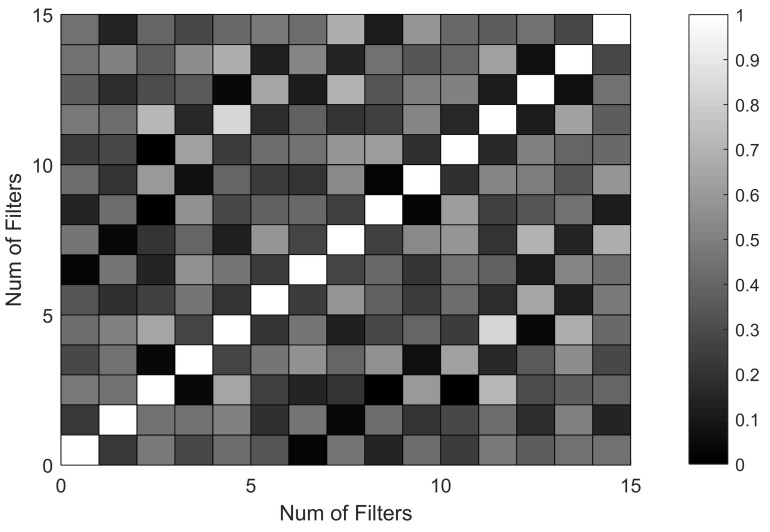
Correlation coefficients of learned filters in Conv1.

To investigate what the monitoring network learned, the first 16 convolution kernels are studied. Figure 6 shows the amplitude spectrums of the convolution kernels. It can be found that the components of different kernels are similar; some of their spectrums have the common peaks, like 8192 Hz, 12.3 KHz, 16.4 KHz, 20.5 KHz, and 28.7 KHz. This proves that the monitoring network successfully extracts the signal features, as these components are also found in the original signal in our previous studies. Then, the correlation coefficients between different convolution kernels are calculated to prove the rationality of parameter setting of the monitoring network, as illustrated in Figure 7. The gray scale reflects the correlation degree, in which the black color (correlation coefficient is zero) means that the convolution kernels are independent, and the white color (correlation coefficient is one) means complete correlation. It can be seen that most of the correlation coefficients are low, except a few ones (for example, the correlation coefficient between No.5 convolution kernel and No. 12 convolution kernel). This indicates that all the useful information for the pulse sound signal recognition is extracted by the first 16 kernels with little repeated information.

**Figure 8:**
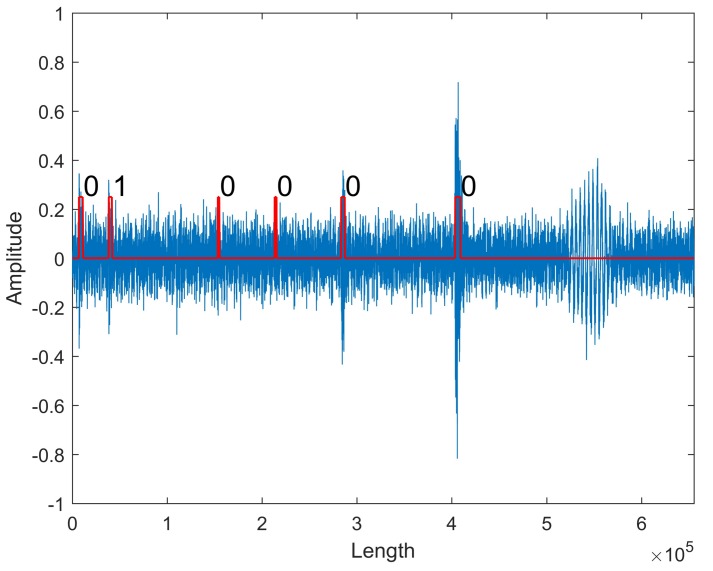
The recognition on original data. The rectangular pulses in red show the positions of pulse sound signals extracted by the intelligent monitoring network. The fault signal is denoted with 1, and the normal signal is denoted with 0.

Finally, the original data that were recorded for 10 h in the industry field is processed with the trained network successfully, which confirms that the proposed method is capable of real-time signal processing and recognizing. Figure 8 shows a section of data flow that is taken for demonstration. Six pulse sound signals are extracted, and the second pulse sound signal is identified as the fault signal correctly.

## 5. Conclusions

In this paper, an intelligent monitoring network is proposed for monitoring the cracks of the anvils of the high-press apparatus used in synthetic diamond industry. This monitoring network realizes real-time monitoring by analyzing the extracted pulse sound signals with CNN technology. In the process of the design and implementation of the network, the SPP-Layer is adopted to transform the variable length input into fixed length output via pooling operations, and an adaptive weighted algorithm for loss function is proposed to solve the class imbalance problem by giving corresponding weight to the loss function of different fault signal sample, which reflects the distribution of feature parameters. The experiments prove that the method proposed in this paper offers high recognition accuracy on the pulse sound signal and realizes the real-time monitoring of the cracks of anvils of high-press apparatus. Furthermore, the structure of the intelligent monitoring network is simple, and the monitoring network can also be used in the recognition of similar pulse sound signals under different applications with a slight modification.

## Figures and Tables

**Table 1:** The Structure of Convolutional Network.

Layer	Conv1	Pool1	Conv2	Pool2	Conv3
Length	N	N/2	N/8	N/16	N/64
Num. of Filters	16	16	64	64	256
Filter Size	64	4	32	4	16
Stride	2	4	2	4	2

**Table 2:** The Structure of SPP-Layer and Fully Connected Layer.

Layer	SPP-Layer	FC1	FC2
Len of Input	(N/64) × 256	16 × 256	256
Len of output	(8 + 4 + 4) × 256	256	2

**Table 3:** The recognition of the network.

Times	Acc (%)	Acc-N (%)	Acc-F (%)
1	99.3	99.5	93.3
2	99.1	99.4	90.0
3	99.1	99.4	90.0
4	99.3	99.6	90.0
5	99.3	99.5	93.3
6	99.1	99.4	90.0
7	99.4	99.7	90.0
8	99.1	99.4	90.0
9	99.2	99.4	93.3
10	99.3	99.6	90.0
Average	99.2	99.5	91.0
